# On-going issues regarding biofilm formation in meat and meat products: challenges and future perspectives

**DOI:** 10.1016/j.psj.2024.104373

**Published:** 2024-10-01

**Authors:** Humaun Oliulla, Md Furkanur Rahaman Mizan, Iksoon Kang, Sang-Do Ha

**Affiliations:** ⁎ Department of Food Science and Biotechnology, GreenTech-based Food Safety Research Group, BK21 Four, Chung-Ang University, Anseong, Gyeonggido, 17546, Republic of Korea; † Department of Animal Science, California Polytechnic State University, San Luis Obispo, CA, 93407, USA

**Keywords:** biofilm, meat product, food industry, food safety

## Abstract

The meat industry has been significantly threatened by the risks of foodborne microorganisms and biofilm formation on fresh meat and processed products. A microbial biofilm is a sophisticated defensive mechanism that enables bacterial cells to survive in unfavorable environmental circumstances. Generally, foodborne pathogens form biofilms in various areas of meat-processing plants, and adequate sanitization of these areas is challenging owing to the high tolerance of biofilm cells to sanitization compared with their planktonic states. Consequently, preventing biofilm initiation and maturation using effective and powerful technologies is imperative. In this review, novel and advanced technologies that prevent bacterial and biofilm development via individual and combined intervention technologies, such as ultrasound, cold plasma, enzymes, bacteriocins, essential oils, and phages, were evaluated. The evidence regarding current technologies revealed in this paper is potentially beneficial to the meat industry in preventing bacterial contamination and biofilm formation in food products and processing equipment.

## INTRODUCTION

As the global population and economic strength of developing nations increase, the consumption of meat and meat-based foodstuffs is predicted to increase considerably (D'Angelo et al., 2018; Li et al., 2020; Ong et al., 2021). Meat is produced from domesticated cattle, swine, sheep, horses, camels, goats, rabbits, and chickens. Because meat and its products provide all the necessary nutrients for humans, such as protein, vitamins, and minerals, they are important for human health. Notably, 30% of the zinc consumed by humans is derived from meat and meat-based products (Pal et al., 2018). It has recently been suggested that vegetables cannot replace some protein and vitamins (particularly B12) found in meat (Bradeeba and Sivakumaar, 2013). Over the past 10 years, it has been reported that the pathogens present in food, including the pathogenic and saprophytic bacteria that frequently cause meat spoilage, can persist as biofilms on the surfaces of foodstuffs and parts of processing facilities (Rahman et al., 2022). Meat spoilage can be triggered by lipid oxidation and autolytic enzymatic reactions, as well as by microorganisms (Addis, 2015). Several foodborne pathogens, including *Escherichia coli* O157:H7, *Salmonella, Campylobacter*, and *Listeria monocytogenes*, can form biofilms in several parts of food-processing facilities (floors, walls, and pipes) and on various materials (stainless steel [**SS**], aluminum, rubber, plastic etc.) (Sofos and Geornaras, 2010). Failure to regulate microorganisms in 1 or more phases of food production can potentially lead to bacterial contamination of foodstuffs (Pal et al., 2018).

Numerous bacterial types develop a matrix of extracellular polymeric substances (**EPS**) called biofilm that is tightly attached to living or inert objects (Abdallah et al., 2014). In addition to causing food degradation and apparatus malfunction, biofilm allows dangerous bacteria to proliferate (Alvarez-Ordonez et al., 2019; Yuan et al., 2020). Therefore, meat and its derivatives should be consistently produced, packaged, stored, and supplied with care because they are highly perishable and frequently contaminated by microorganisms (Heetun et al., 2015; Oliulla et al., 2024). Meat and meat products potentially spread *L. monocytogenes*, and several foodborne outbreaks have caused tremendous public health concerns (Barcenilla et al., 2022). Meat and poultry are carriers of 30.9% of foodborne illnesses, resulting in up to USD 20.3 billion in economic losses (46.6% of total costs) in the United States between 1998 and 2017 (Scharff, 2020). *Salmonella* and *Campylobacter* are the most frequently detected pathogens in chicken and turkey (Chai et al., 2017; Rouger et al., 2017).

Therefore, hygiene monitoring in all aspects of meat production is essential for identifying and interpreting the overall patterns of biofilm formation. In this paper, we highlight several intervention technologies that are employed to control pathogens and inhibit biofilm development in meat and its products.

## HAZARDS IN MEAT PRODUCTS

Meat and meat-based food items provide valuable nutrients in the daily diet of humans. The demand for meat as a source of top-quality protein has increased over the past 50 years. However, meat has a short shelf life and is easily spoiled, mainly due to bacterial growth and biofilm formation. Meat spoilage results from complex reactions, such as biological (bacteria, yeast, and mold), enzymatic (lipases and proteases), chemical (browning and oxidation), and physical (freezing, drying, and pressure) processes (Casaburi et al., 2015; Iulietto et al., 2015). Although meat deteriorates owing to multiple factors, bacteria (biofilm) represent the most frequent cause of quality loss in animal-derived foods (meat products). Meat and meat products are vulnerable to extensive cross-contamination, and pathogens come from the gastrointestinal tracts of animals, conveying during processing to the improper storage of finished goods. Table 1 displays examples of major foodborne infections caused by biofilm formation on meat surfaces.

**Table 1:** Examples of epidemics caused by significant foodborne pathogens capable of forming biofilm on meat and related surfaces.

Responsible pathogens	Products categories	Morphology	Biofilm formation	Pathogenicity	References
*Listeria* *monocytogenes*	Ready-to-eat meat, cured or uncured beef, SS surface	Gram-positive, rod-shaped, diameter of 0.5-4 μm ×0.5-2.0 μm, noncapsulated.	*L. monocytogenes* is capable of forming biofilm on meat and related surfaces. *L. monocytogenes* biofilm on SS surfaces ranged from 10^4^ to 10^5^ CFU/cm^2^.	Flu-like indications in healthy adults. Diarrhea, fever, vomiting, joint pain, and headache in children. Pregnancy-related miscarriage and stillbirth.	Belessi et al. (2011), Han et al. (2016), Veasey and Muriana (2016), Mohamed et al. (2022)
*Escherichia coli*	Beef, Pork, SS, polyvinyl chloride (PVC)	Gram-negative (diameter 0.25-1.0 μm × 2.0 μm), non-spore-forming, usually motile by peritrichous flagella.	Strong biofilm-forming ability of *E. coli* O157:H7 in beef extract (7.88 log CFU/mL) and high sanitizer resistance. Biofilm formation on materials (SS, PVC) commonly used in the meat industry.	Hemorrhagic colitis, hemolytic uremic syndrome, vomiting, diarrhea, and bloody diarrhea.	Croxen et al. (2013), Wang et al. (2016), Liu and Liu (2020), Liu et al. (2021)
*Staphylococcus aureus*	Pork	Gram-positive, round-shaped, diameter of 0.5–1.0 μm	*S.* aureus had moderate or strong biofilm production capability. After 4 d of storage, the total viable count in the control group reached to 5.8 log CFU/g in pork.	Diarrhea, vomiting, and even toxic shock symptoms	Foster (1996), Archer (1998), Kadariya et al. (2014), Miao et al. (2015), Dai et al. (2019)
*Salmonella*	Poultry, Beef, Pork, Fermented and dried meats	Gram-negative (diameter 2–5 µm × 0.5–1.5 µm), non-spore-forming, rod-shaped, and facultative anaerobic, peritrichous flagellum	Form biofilm in meat-processing environment and serve as a source of cross-contamination. Average biofilm formation of *Salmonella enterica* ser. Enteritidis on chicken skin was 6.07 log CFU/g	Abdominal pain, nausea, vomiting, fever, and typhoid fever	Mor-Mur and Yuste (2010), Wang et al. (2013), Gut et al. (2018), Mutz et al. (2019), Byun et al. (2022)
*Campylobacter jejuni*	Pork, Poultry	Gram-negative, S-shaped rods (0.2–0.8 μm wide and 0.5–5.0 μm long), nonsaccharolytic, nonspore forming	Biofilms play a significant role in the prevalence and transmission of *C. jejuni.* *Campylobacter* colonizes the gut of the infected chicken at levels of 10^8^ - 10^9^ CFU/g of caecal contents and during processing, can result in contamination of the raw product.	Clinical manifestations of campylobacteriosis are extremely diverse, ranging from a complete absence of symptoms to fulminating sepsis and rarely death, mainly in immune-susceptible hosts.	Cawthraw et al. (1996), Solow et al. (2003), Hanning et al. (2008), Snelling et al. (2005), Silva et al. (2020), Thomas et al. (1998)
*Pseudomonas* spp.	Meat surface, Polycarbonate (PC)	Gram-negative (0.5–0.8 μm × 1.5–3.0 μm), aerobic, single polar flagellum	The biofilms of P. aeruginosa presented a bacterial biomass of 7.8 log CFU/cm^2^ on PC surface. By d 3 (76 h), biofilms had started to form covering most of the meat surface (10^4^ to ∼10^8^ CFU/cm^2^).	Some patients get gastroenteritis, causing diarrhea and or bleeding.	Abdallah et al. (2015), Hoff et al. (2020), Wu and Li (2015)
*Brochothrix thermosphacta*	Poultry meat	Gram-positive, rod-shaped, nonmotile, non-spore-forming, facultative anaerobic	*B. thermosphacta* inoculated with about 5–6 log CFU of attached bacteria per cm^2^ on pork adipose tissue disks and got reductions of 0.84 log cycles after treatment.	Causes meat spoilage which may lead to food-poisoning-related diseases.	Kilcher et al. (2010), Korber et al. (2002), Stanborough et al. (2017), Rouger et al. (2018)
*Carnobacterium* spp.	Vacuum-packaged chicken meat, Screw conveyor	Gram-positive rods belonging to the family *Lactobacillaceae*.	*C. maltaromaticum* can form biofilm (about 8.7 log CFU/cm^2^) in the meat-processing environment.	Fever, malaise, and pain in the neck and head	Hoenigl et al. (2010), Lawson and Caldwell (2014), Rouger et al. (2018), Wagner et al. (2021)

## MODE OF ACTION OF BIOFILM FORMATION

In meat-processing facilities, the environment is conducive to supporting the survival and proliferation of microorganisms on food-contact surfaces, including knives, tables, and conveyor belts. If biofilms are formed, removing them may not be possible when basic cleaning and disinfection techniques are used (Giaouris et al., 2014). Microbial cells' natural survival strategy entails constructing a biofilm on a surface for successful interactions with other cells in the biofilm to obtain a fitness advantage by providing an escape from competition for nutrients, space, and defense against adverse environmental conditions. The surface could be biotic (e.g., meat, skin, fruit, intestines, urogenital tract, or the oral cavity) or abiotic (ground, ceilings, pipes, utensils, or surfaces that come into contact with food) (Bai et al., 2021). Because a meat surface has abundant nutrients, biofilms can grow rapidly on the surface. However, the biofilm-formation process, followed by microbial bonding, is complex and depends on multiple variables, including environmental factors (such as pH, temperature, and nutrients), the physical qualities of products, and the strain of the biological agent (Dhivya et al., 2022). The presence of glucose, lactic acid, amino acids, and nitrogenous substances can be used as a source of energy for microbial development and deterioration of meats. Glucose is primarily catabolized during the first stages of microbial development under both aerobic and anaerobic conditions. After the exhaustion of glucose compounds, lactic acid is used as a secondary nutrient source (Nychas et al., 2008; Bekhit et al., 2021). The eventual pH of meat is affected by the degradation of glycogen deposits in muscles, which determines the rate of fresh meat damage by bacteria (Bekhit et al., 2021). Typically, meat deterioration is driven by the abundance of meat-surface bacteria.

**Figure 1:**
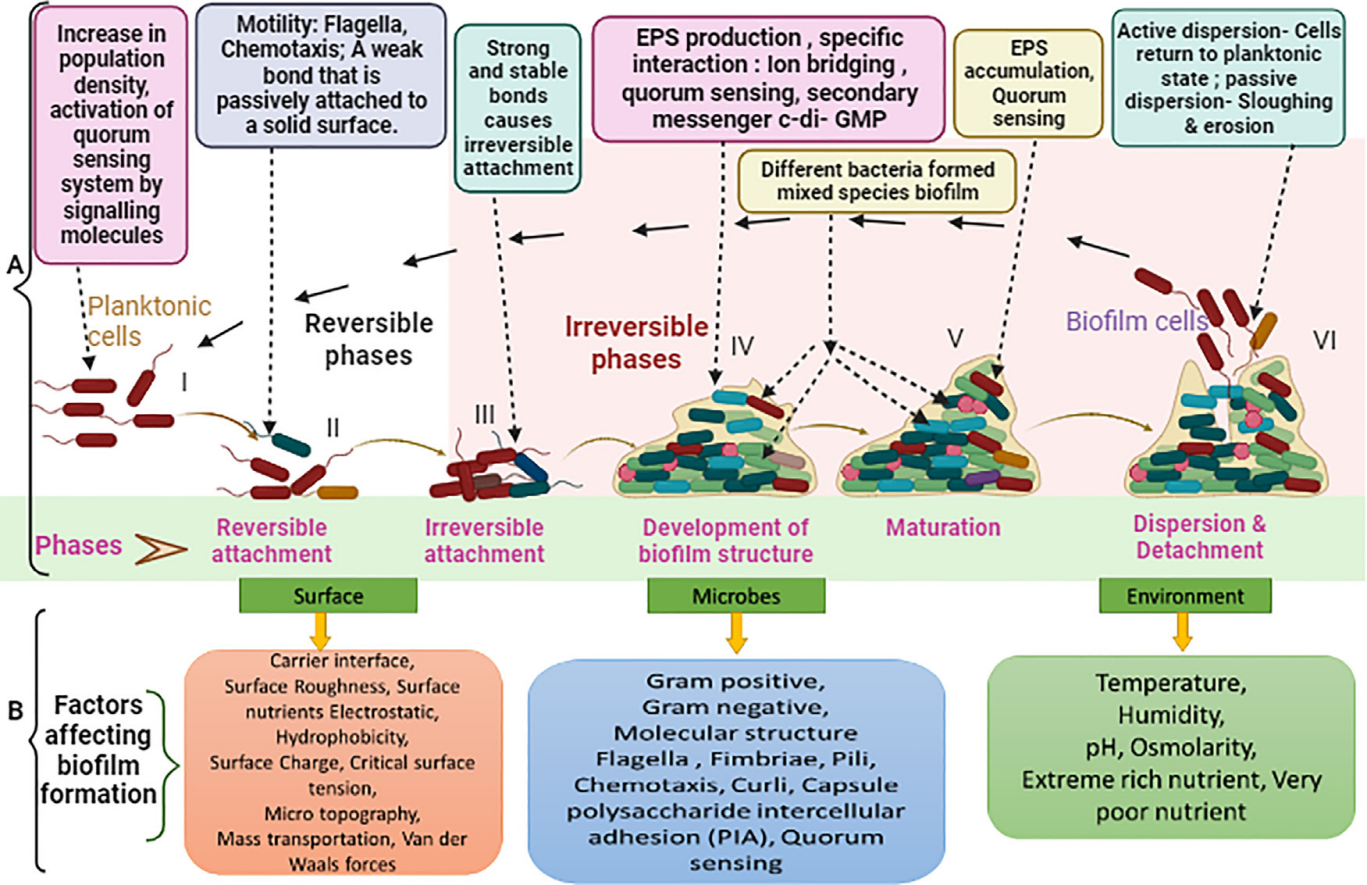
(A) Stages of biofilm development. (I) Increase in population density, activation of QS system by signaling molecules (Gram-positive bacteria: autoinducing peptide [API]-mediated QS phosphorylation cascade; Gram-negative bacteria: autoinducer-1 [AI-1] acylated homoserine lactone [AHL], autoinducer-3 [AI-3]; Gram-positive and Gram-negative: autoinducer-2 [AI-2]). (II) Motility (flagella and chemotaxis) allows for active movement to the point of attachment. Ability of the appendages to attach to a cell surface to overcome the cell membrane's repelling forces. A weak bond that is passively attached to a solid area (electrostatic connections, van der Waals forces, and hydrophobic interactions). (III) Strong and stable bonds cause irreversible attachment (hydrogen bonding, dipole-dipole interactions, and hydrophobic and ionic covalent bonding), cell-to-cell communication, surface charge, AHLs, and processed oligopeptides. (IV) Cell aggregation and extracellular polymeric material synthesis occur simultaneously (EPS), QS, and specific interaction. (V) Entrenchment of numerous-cell-thick cell clusters in the biofilm matrix indicates biofilm maturation, and subsequently, the biofilm fully develops into micro-colonies. (VI) Active dispersion of biofilm cells commences through QS-controlled genetic materials, and cells revert to their planktonic state. Passive dispersion of biofilm cells under force. Inhibition of c-di-GMP signaling pathways. Inherent cell properties (mainly autolysis) (Coughlan et al., 2016; Muhammad et al., 2020). (B) Biofilm formation owing to dynamic relationships among various components (mainly, 3 groups of factors).

A biofilm is an association of microorganisms that reside in an external polymeric structure and adhere to each other on living or nonliving surfaces. Biofilm cannot be eliminated without proper surface washing (Jamal et al., 2018). Although biofilm formation is an intricate process, scientists agree that it can be categorized into a few basic steps, such as surface adhesion, micro-colony formation, biofilm-infrastructure maturation and development, and bacterial dispersion (Figure 1) (Jamal et al., 2018)**.** A wide range of both internal and external conditions, such as environmental factors (pH, humidity, nutrients, and temperature) and microbiological variables (microbial taxonomy, morphology, Gram-negativity/positivity, molecular structure, and virulence factors), interact to influence biofilm formation and operation, potentially affecting a microbe's capacity to build biofilm (Figure 1) (Whitehead and Verran, 2015; Sharan et al., 2022). Thus, it is indispensable to develop effective approaches to control and eliminate the formation of biofilm in the meat industry.

## CURRENT INTERVENTIONS TO PREVENT BACTERIAL CONTAMINATION AND BIOFILM FORMATION

### Ultrasound

**Figure 2:**
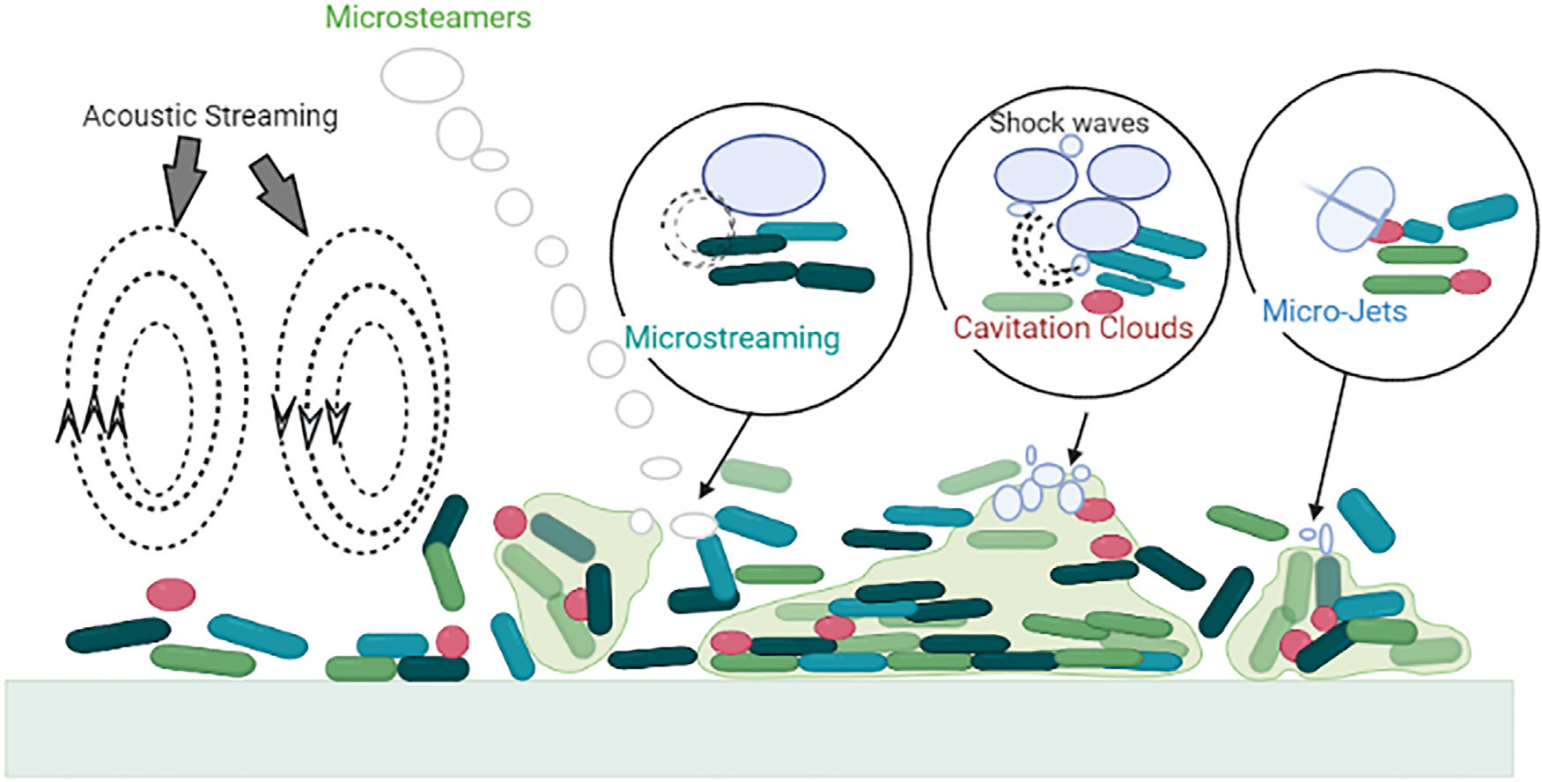
Schematic illustrating the various mechanisms by which cavitation can remove a mechanical biofilm. The direction of ultrasonic sonication is indicated by the black arrow. As illustrated, when a biofilm is "soft," micro-jets point away from it, whereas when it is "rigid," they extend toward it (e.g., mineralized biofilms) (Vyas et al., 2019). Micro streamers are ribbons of cavitating microbubbles, and cavitation clouds are clusters of cavitating bubbles.

One of the well-known nonthermal techniques is ultrasound (**US**), and it has been extensively explored in the food industry in the areas of microbial control, curing, and cleaning (Turhan et al., 2013; de Sao Jose et al., 2014; Cao et al., 2018). US is a form of kinetic energy that can produce shear forces to remove biofilm from food and equipment surfaces (Liu et al., 2022). Ultrasonic wave disruption of biofilms is associated with the bubble and fluid flow phenomena that occur during sonication (Figure 2).

Three major factors that influence the antibiofilm effect of US are the nature of the surface, the exposure period, and the US intensity (Birmpa et al., 2013). Erriu et al. (2014) demonstrated that higher levels of biofilm structure disintegration and cell separation could be achieved using high-intensity ultrasound (HIU: over 5 W/cm^2^), which delivers adequate mechanical energy, whereas low-intensity ultrasound (LIU: up to 3 W/cm^2^) stimulated bacterial metabolism, leading to the development of a biofilm that was more durable and strongly attached to the surface. The amounts of biofilm removed at different power levels differed significantly. It was noted that LIU (2 W/cm^2^) did not significantly reduce the bacterial viability of the biofilm. To eliminate surface-attached biofilms, the transient cavitation effect occurring at HIU (>10 W/cm^2^) is necessary (Erriu et al., 2014). However, even at a high-power level, the bactericidal effect of US is limited (Koibuchi et al., 2018). Yu et al. (2021) reported that when the acoustic pressure on the surface of the glass side-cultivated *Staphylococcus aureus* biofilm was –1.38 × 10^5^ Pa, the application of HIU (240 W) removed up to 96.02% of biofilm, although the inactivation rate remained at 53.23%. Low-frequency sonication reduces bacterial viability in biofilms more effectively than high-frequency sonication (Erriu et al., 2014; Chemat et al., 2017). In addition to the US treatment parameters, the bacterial strain significantly influences the bactericidal effect of HIU. The application of US treatment to *L. monocytogenes* biofilms has been found to result in varying degrees of bactericidal activity. Torlak and Sert (2013) observed the efficacy of low-frequency US (35 kHz) in removing *L. monocytogenes* biofilms from a polystyrene surface and detected 20%, 45%, and 87% reductions in biofilm density after 1-, 5-, and 15-min treatments, respectively. Furthermore, after US treatment for 1 and 4 s, Morild et al. (2011) reported that the total viable bacterial counts on pork jowl skin and meat decreased by 1.1 log and 3.3 log CFU/cm^2^, respectively. The extent of decrease on the skin ranged from 1.7 log to 3.3 log CFU/cm^2^, which was significantly greater than the 1.1 log to 2.5 log CFU/cm^2^ on the meat portion. Ultimately, longer treatment time resulted in greater reductions. The bactericidal effect and biofilm removal of high-intensity and low-frequency US is mediated by the following mechanism: the basic structure of biofilm is partially destroyed by mechanical oscillation as a physical mechanism, altering the integrity of cells and leading to the separation of biofilm and fatal effects on microbes.

The total elimination of foodborne microorganisms may not be possible with individual nonthermal technologies. Numerous studies have recommended the combined effects of US and other strategies (e.g., antibacterial agent, heat, high pressure, chemical disinfectant) to enhance the control of viable cells (Baumann et al., 2009; Shao et al., 2020). Conveyor belts, cutting boards, and meat trays (polystyrene) are some of the surfaces used in the meat industry that come into contact with meat and meat products. In a study, it was found that *L. monocytogenes* biofilm on polystyrene was resistant to benzalkonium chloride (400 mg/L), which only eliminated 60% of viable cells after 15 min, whereas the combination of US (35 kHz) and benzalkonium chloride (100 or 400 mg/L) resulted in a reduction of viable cells that was greater than 90% or 95% (Torlak and Sert, 2013). Similarly, *S. aureus* biofilm on SS surface was significantly reduced by about 3.7 log CFU/cm^2^ as a result of the combination of acidic electrolyzed water and US for 20 min (Shao et al., 2020). These synergistic effects are due to enhanced penetration and diffusion of chemical disinfectants through the microbial biofilms. According to Baumann et al. (2009), combined treatment with US and ozone effectively removed the *L. monocytogenes* biofilm that had grown on SS, which is regularly used in the meat industry. In addition, viable *L. monocytogenes* biofilm cells were reduced by up to 7.31 log/mL after combined treatment with US (60 s) and ozone (0.50 ppm). These findings highlight the potential of the combined applications of US and ozonation or electrolyzed water for sanitizing meat surfaces as well as food equipment.

The use of US for controlling bacterial biofilms has increased recently. Chemical disinfectants, chelating agents, enzymes, and other techniques could be combined with sonication or ultrasonication to produce a synergistic effect on biofilm elimination.

### Cold Plasma

**Figure 3:**
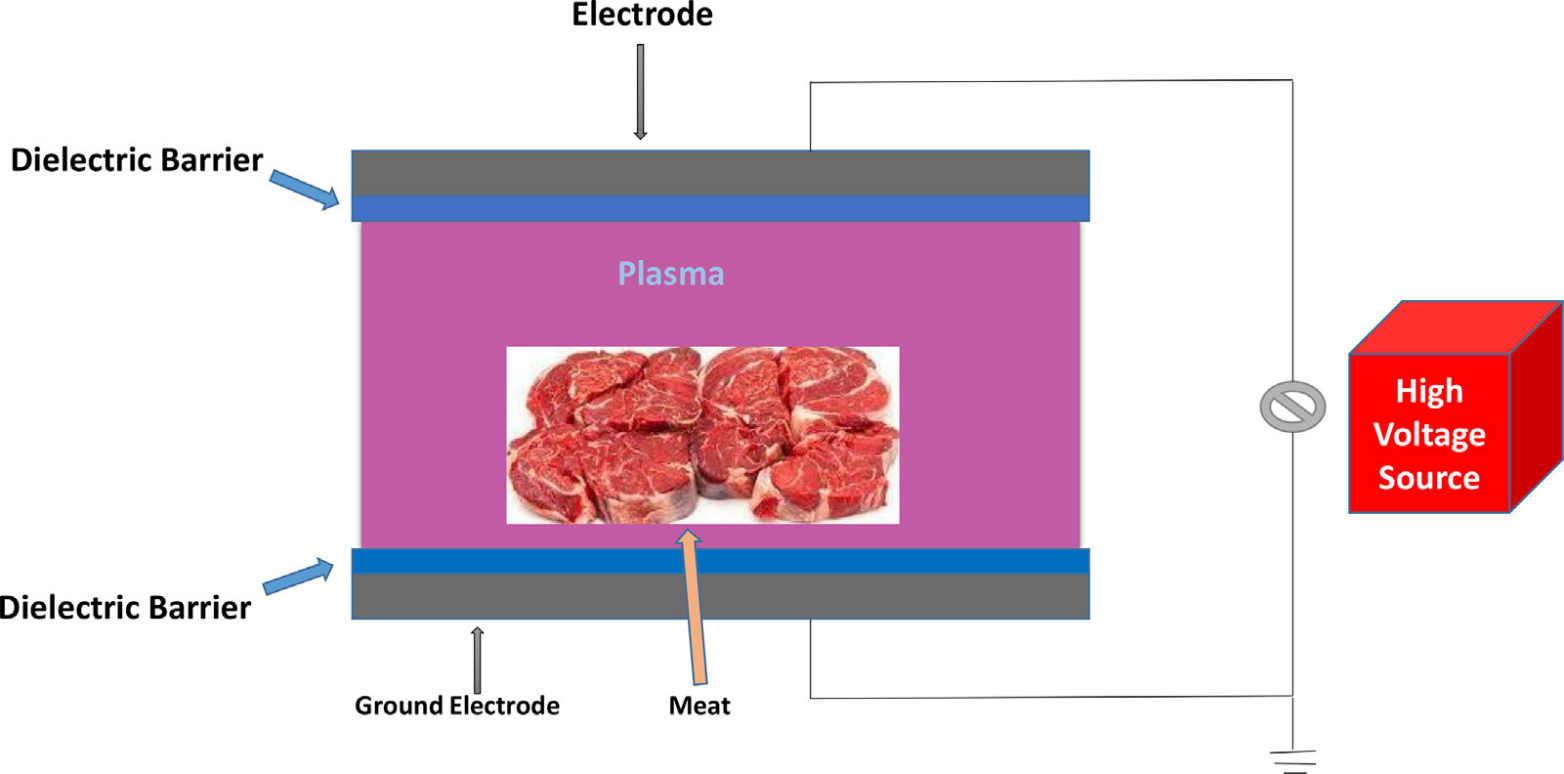
Illustration of a DBD-based atmospheric cold-plasma meat-processing system (Akhtar et al., 2022).

One of the most cutting-edge and emerging technologies is cold plasma (**CP**), and its ability to improve microbial safety in raw and processed meat products has been extensively investigated (Kim et al., 2014; Yong et al., 2014; Chaplot et al., 2019). CP can be formed by introducing gas (air or any other gas combination) between electrodes and employing dielectric barrier discharge (**DBD**), radiofrequency, and microwave power sources to generate a significantly strong electric field (Nasiru et al., 2021; Akhtar et al., 2022). DBD and atmospheric pressure (**AP**) plasma jets are the 2 most popular CP devices utilized in the meat-processing industry due to their simple design and adaptability to a range of treatment requirements. Two electrodes running at various voltages and divided by a dielectric medium generate barrier discharges (Nasiru et al., 2021). The inter-electrode gap can be used for gas ionization because the dielectric barrier between the electrodes prevents electric discharge and limits current passage (Nasiru et al., 2021). Depending on the electrode and barrier configurations, a DBD can be produced, as explained by Akhtar et al. (2022). Figure 3 shows a schematic view of meat processing using atmospheric CP and DBD.

**Figure 4:**
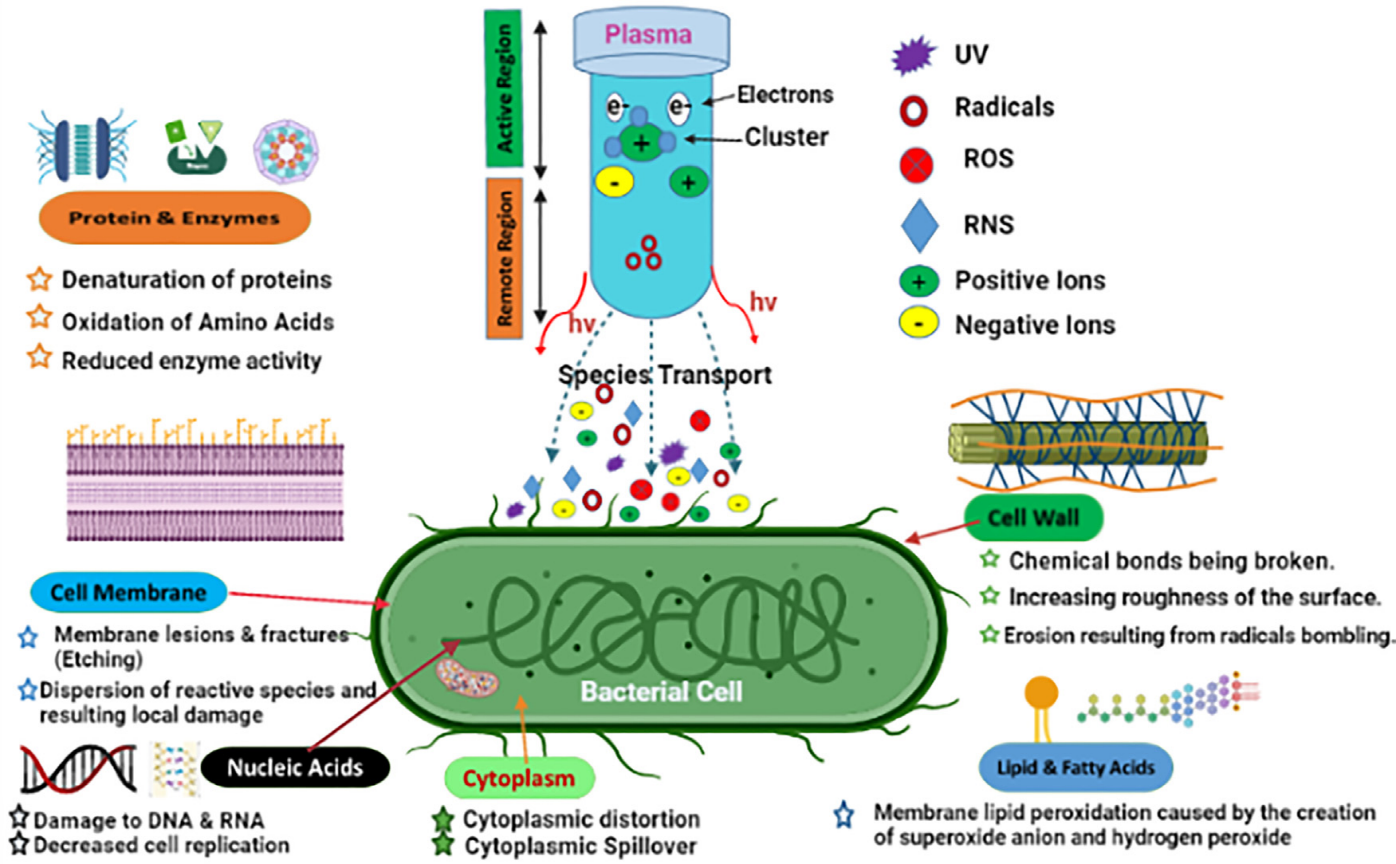
Illustration of the effect of cold plasma on bacterial-cell architecture (Misra and Jo, 2017; Akhtar et al., 2022).

CP comprises considerably reactive and excited atoms, molecules, electrons, positive and negative ions, free radicals, gas atoms, and photons (Dhivya et al., 2022). Most importantly, CP can produce a wide range of highly reactive oxygen species and reactive nitrogen species. These reactive species are used to remove biofilms through various biochemical processes (Bourke et al., 2017). Generally, depending on the interactions between cells and the plasma ions, different microbial cells respond differently to plasma treatment. Oxidation is caused directly by a reaction between elements in the plasma composition and the surfaces of microbial cells. It is also widely acknowledged that plasma damages the DNA of microorganisms. CP's ability to suppress biofilm growth is strongly influenced by several factors, such as the CP device, the microbial features, the biofilm density, the input-gas composition, the proximity between the ion source and biofilm, the biofilm maturity, and the material-surface characteristics. There is substantial evidence showing the antibiofilm effect of CP and the role of CP in reducing the pathogenicity of harmful microorganisms and bacterial spores (Ziuzina et al., 2015). Kim et al. (2013) demonstrated that owing to its outstanding microbicidal efficacy and low process temperature (60°C), CP has gained prominence in food-processing facilities, particularly in the meat and poultry industries. As shown in Table 2, numerous research studies have demonstrated its efficacy for the safety of various meat and meat products, including fresh meats (beef loin, pork shoulder, and chicken breast) (Bae et al., 2015), beef (Kim et al., 2014), beef jerky (Yoo et al., 2021), chicken (Dirks et al., 2012), raw poultry meat (Chaplot et al., 2019), pork (Huang et al., 2019), and processed meat products (Lee et al., 2018). Liu et al. (2016) reported that reactive charged particles are important because they can severely injure microbial cells. A graphical depiction shows the specific mechanism by which CP interacts with bacteria (Figure 4).

**Table 2:** Applications of CP on microbial elimination on meat.

Meat products	Parameters	Target microbes	Major findings	References
Beef jerky	Atmospheric pressure plasma, 0.05% clove oil, ET: 4 min, gas: air, V: 8.4 kV, f: 2.2 kHz	*Escherichia coli* O157:H7	Decline of >7.5 log CFU	Yoo et al. (2021)
Beef	f: 20 kHz, V: 6 kV, ET: 0.5 to 10 min.	*E. coli*	After 2- and 5-min treatments, there was a reduction of 0.9 log and 1.82 log CFU/cm^2^, respectively.	Stratakos and Grant (2018)
Ready-to-eat ham	f: 2 and 10 kHz, V: 6.4 or 10 kV. ET: 10–20 min at 22°C	*Salmonella enterica* ser. Typhimurium and *Listeria monocytogenes*	The maximal *S.* Typhimurium inhibition was 1.14 log. *L. monocytogenes* was decreased by 1.02 log.	Lis et al. (2018)
Boneless skinless chicken breast and chicken thigh with skin	DBD plasma, V: 30 kV, Current: 1 Amp, ET: 3 min	*S. enterica* *Campylobacter jejuni*	Reduction values of 1.3 log to 1.08 log CFU/g on the skin and approximately 2.5 log CFU/g on the breast. Reduction values of 1.4 log to 3.1 log CFU/g on the skin and nearly 2.5 log CFU/g on the breast	Dirks et al. (2012)
Chicken breast meat	Internal CP: V: 60–80 kV ET: 5 min, kept at 4°C for 5 d. DBD-CP in-package: V: 100 kV, ET: 1-4 min	*Campylobacter* and *Salmonella* Natural microflora	Reduction of *Campylobacter* and *Salmonella* by 1.11 log and 1.05 log CFU/mL, respectively. Reduction of 2 log CFU/g in mesophiles, psychrotrophic, and *Enterobacteriaceae* within 5 min	Zhuang et al. (2019), Moutiq et al. (2020)
Raw poultry	DBD-CP combined with peracetic acid (**PAA**) (100–200 ppm) f: 3.5 kHz, V: 0-30 kV, power: 0-200 W, ET: 1-6 min, distance: 2 mm	*Salmonella*	In comparison to **PAA** or CP treatments, the combined therapy resulted in decreases of 2.3 log to 5.3 log CFU/cm^2^.	Chaplot et al. (2019)
Fresh and Frozen Pork	Air, f: 58 kHz, V: 20 kV, current: 1.5 A, ET: 0-120 s, plasma jet	*E. coli* O157:H7 and *L. monocytogenes*	1.5 log reduction in *E. coli* O157:H7 and > 1.0 log reduction in *L. monocytogenes*.	Choi et al. (2016)
Bacon	DBD plasma, helium/oxygen, power: 125 W, ET: 90 s	*L. monocytogenes* *E. coli* and *S.* Typhimurium	Decrease of 2.6 log CFU/g. Decrease of 3.0 log CFU/g. Decrease of 1.7 log CFU/g.	Kim et al. (2011)

Note: f , frequency; V, voltage; ET, exposure time.

Numerous physiological functions can be controlled via quorum sensing (**QS**) mechanisms, including biofilm development and the expression and dissemination of microbial pathogenicity. Plasma potentially inhibits the activation of virulence genes and restrains QS signal molecules. In an analysis of an *Enterococcus faecalis* biofilm, Li et al. (2019a) observed that plasma-activated water successfully prevented biofilm formation while lowering the expression of pathogenic genes associated with QS. Chaplot et al. (2019) provided evidence that atmospheric CP decreased the *Salmonella enterica* ser. Typhimurium count in chicken meat by 5.3 log CFU/cm^2^. When *Candida albicans, Pseudomonas aeruginosa*, and *S. aureus* biofilms were treated using an argon plasma jet, the biofilms were more vulnerable to the plasma compared to the planktonic organisms (Taghizadeh et al., 2015). This ability of CP to control virulence factors and molecules involved in QS signaling makes it feasible to use CP for antibiofilm treatments in the meat industry.

As mentioned above, a single nonthermal technology may not be adequate to inactivate foodborne microorganisms completely and may even cause a microbial stress response, resulting in a sublethally damaged state or viable but nonculturable condition (Liao et al., 2020; Wu et al., 2020). Different hurdle interventions expose microbial cells to multiple stressors and cumulative adverse effects, lowering the possibility of the development of stress resistance. The hurdle technique of CP combined with 100 and 200 ppm peracetic acid (**PAA**) resulted in the inactivation of *S.* Typhimurium populations in raw poultry by 3.8 log and 5.3 log CFU/cm^2^, respectively (Chaplot et al., 2019). In a different study, the combination treatment of CP and lemongrass oil resulted in the inactivation of *L. monocytogenes* in poultry loin by 2.80 log CFU/g (Cui et al., 2017). According to Govaert et al. (2019), the biofilms of *L. monocytogenes* and *S.* Typhimurium on polystyrene Petri dishes (meat contact surface) were almost completely inactivated by a combination treatment of cold atmospheric plasma and hydrogen peroxide (H_2_O_2_; 0.05% and 0.2%, v/v), which resulted in reductions of up to 5.42 log and 5.90 log CFU/cm^2^, respectively. The bactericidal effect of the combined treatment of clove oil (0.05% concentration) and encapsulated AP (exposure time: 4 min, gas: air, voltage: 8.4 kV, frequency: 2.2 kHz) plasma was investigated in inoculated beef jerky against *E. coli* O157:H7 (initial microbial count was 8.54 log CFU/mL), resulting in more than 7.5 log reduction (Yoo et al., 2021). It is important to note that the effectiveness of this hurdle technology may vary due to differences in plasma treatment parameters (like the power, time, and gas component), which produce different reactive species. Thus, in order to enhance antimicrobial activity and maintain high food quality, the CP-based hurdle combined with other strategies provides a cumulative reduction of microorganisms.

Although CP treatment has been successful in inactivating foodborne bacteria in raw meat and meat-based products, its disadvantages include accelerating lipid oxidation and negatively affecting sensory attributes. With continuous advancements, CP is generally expected to be a promising meat processing technology with respect to the safety and quality attributes of meat (Misra and Jo, 2017). In summary, the current literature data suggests that CP technology can reduce biofilm formation and bacterial contamination in the meat industry, as well as provide an effective solution to the future antimicrobial challenge.

### Ozonation

Ozonation is a well-established disinfection technique with a wide range of antimicrobial qualities and exhibits effectiveness against Gram-positive and Gram-negative microorganisms on food surfaces (Pandiselvam et al., 2019). Ozone and its use as a disinfectant in the food processing industry have recently attracted considerable interest. Owing to its powerful oxidizing action, ozone can kill bacteria by oxidizing their cell walls, membranes, and other cellular components (Aslam et al., 2020; More and Rao, 2022). In 2001, ozone was formally recognized by the United States Food and Drug Administration (**FDA**) for use in the food industry in direct contact with food items such as meat, poultry, and fish, in addition to being a surface cleanser in food processing plants (Zhang et al., 2016; Al-Qadiri et al., 2019).

Brodowska et al. (2018) reported that ozone affects microorganisms by targeting their cell membranes, cell envelopes, cytoplasm, spore coatings, and viral capsids. Ozone inhibits bacterial growth through 2 main processes: 1) oxidation of sulfhydryl groups and amino acids in enzymes, proteins, and peptides to smaller peptides and 2) oxidation of polyunsaturated fatty acids to acid peroxides, thus eliciting cell death (Zouaghi and Cantalejo, 2016). Although not proven, ozone has been hypothesized to react with cell membrane lipids, generate reactive species, enter the host cell's nucleus, and disrupt its genetic material (Khanashyam et al., 2022).

**Figure 5:**
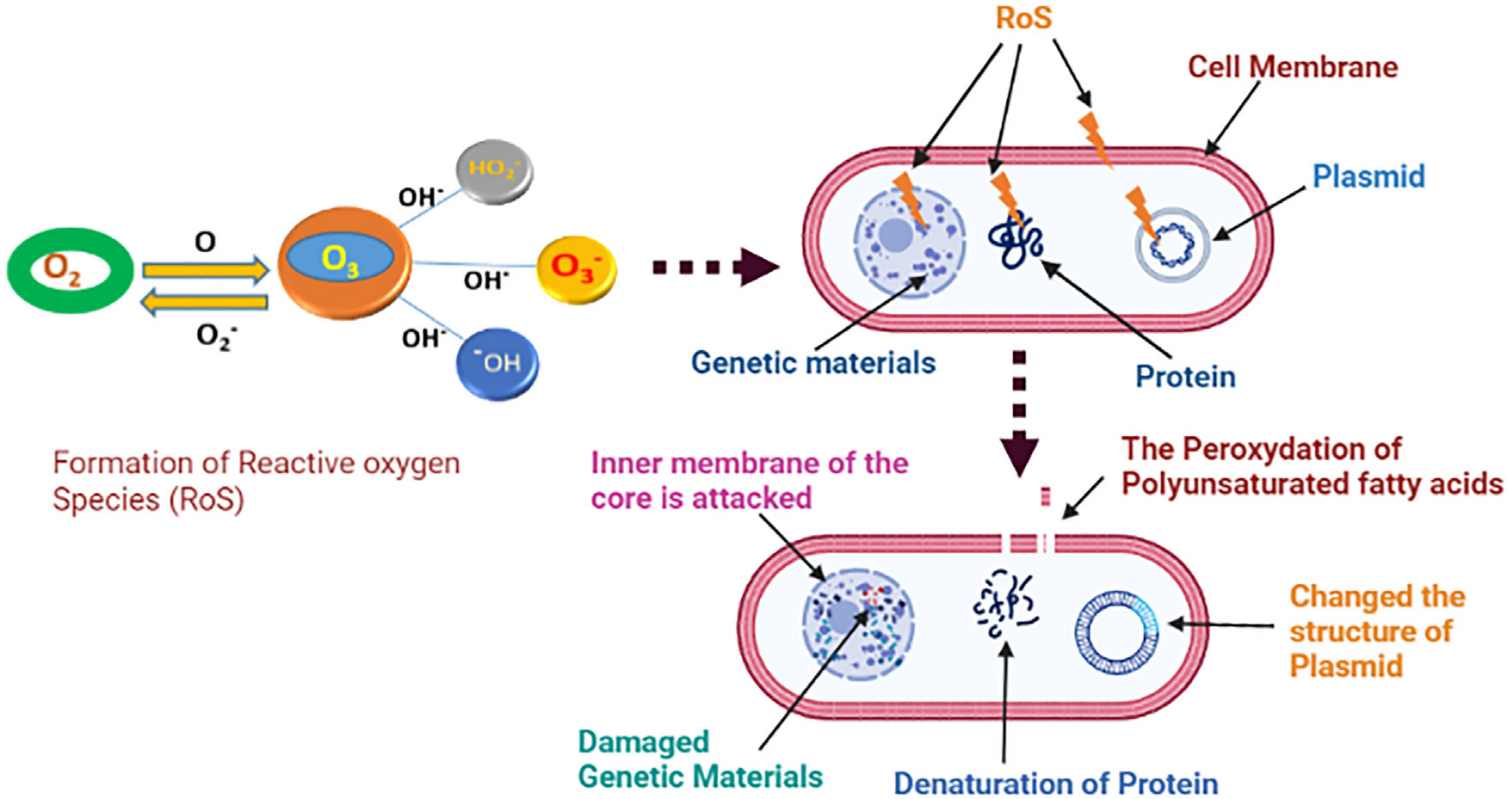
Ozone-induced bacterial inactivation (Khanashyam et al., 2022; Roobab et al., 2022).

The mechanism underlying ozone's decontamination process is depicted in Figure 5. Several studies have explored how ozone prevents microbial biofilm formation. According to Panebianco et al. (2021), ozone inhibits the ability of cells to generate an EPS matrix, induces structural disintegration of the matrix, and diminishes the overall biomass of the constructed biofilms. The safety of meat and meat products is the foremost priority, considering widespread concerns regarding the illnesses caused by virulent microorganisms, such as *Listeria, Salmonella*, and *Campylobacter*. Ozone treatment significantly reduces the number of microbial cells on food products without food quality loss. When ozonated water treatment was applied for 1 min at 1.0, 2.0, and 4.0 ppm, the amount of *L. monocytogenes* single strain biofilm on polystyrene was reduced by ∼0.9 log, 3.4 log, and 4.1 log CFU/cm^2^, respectively (Korany et al., 2018). Marino et al. (2018) reported that the use of gaseous ozone at a concentration of 5 ppm eliminated 3.0 log of microbial biofilms of *Pseudomonas fluorescens* and *S. aureus* in around 17 and 6 min, respectively. The biofilm of *L. monocytogenes* on SS was reduced by 4.2 log CFU/mL when treated with 1.0 ppm ozone for 60 s, as described by Baumann et al. (2009).

The bacterial populations in meat and meat-based foodstuffs can be controlled using ozone as a potent antimicrobial agent. It is nontoxic and does not leave any chemical residues. The sensitivity to ozone is influenced by factors such as its organic content, temperature, physical state, the initial microbial load, and the type of microorganism. The fact that different microorganisms vary in their sensitivity to ozone serves to counterbalance this beneficial property of ozone. Consequently, one of the main challenges for using ozone is determining an optimal ozone dose. It is important to maintain caution when applying high ozone doses to achieve optimal microbial inactivation. In certain instances, prolonged contact times combined with high doses of ozone have impacted the quality parameters of meat and related products because ozone can cause discoloration and lipid oxidation. Furthermore, ozone gas can enter the human respiratory system, thus requiring precautions during ozone handling or exposure (Zhu, 2018). It is crucial to understand the degree of ozonation that can balance microbial decline with meat quality maintenance. As ozone is an extremely unstable gas, care should be taken to ensure sufficient contact time for the removal of ozone-resistant compounds (pesticides) to prevent partial oxidation of the targeted compound. For improved ozone usage, conducting additional research and mitigating consumer concerns regarding sensory aspects, health, and safety are necessary.

### Essential Oils

Efforts to satisfy consumer demands for more natural preservatives have been challenged by the continuous use of synthetic preservatives (Aminzare et al., 2016; Alirezalu et al., 2020). Over 3,000 essential oils (**EO**s) have been identified to date; nevertheless, only 300 EOs are relevant for commercial use (Dima and Dima, 2015). Owing to their diverse biological properties, such as antibacterial, antioxidant, antifungal, and anti-inflammatory properties, EOs may replace synthetic chemicals (Khayyat and Roselin, 2018). Significant research on the antibacterial effect of various EOs with/without encapsulation in meat and meat products has been conducted (Pateiro et al., 2021).

**Figure 6:**
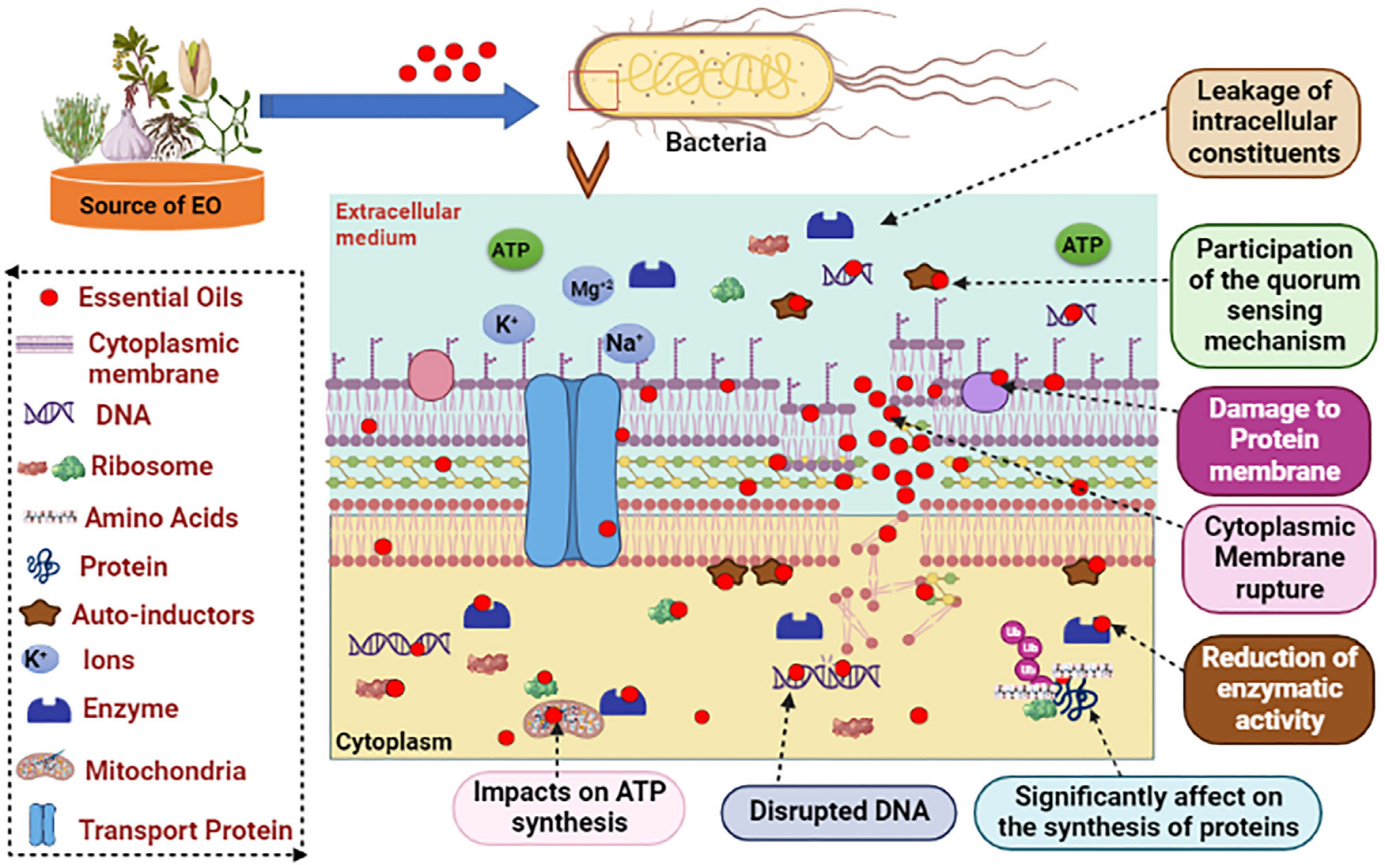
Mechanisms of action of essential oils against microbial cells (Pateiro et al., 2021; da Silva et al., 2022).

In general, Gram-negative bacteria provide greater resistance against the antibacterial activity of EOs than Gram-positive bacteria due to the difference in the cell wall structure (Bharti et al., 2020). Gram-negative bacteria possess a complex cell envelope compromising an outer membrane linked to a thin inner layer of peptidoglycan, which acts as a selectively permeable barrier. The outer membrane contains proteins and hydrophilic lipopolysaccharides and thus shows enhanced resistance to the diffusion of hydrophobic compounds in EO (Behbahani et al., 2019). This contrasts with the hydrophobic layer surrounding Gram-positive bacteria. Comprised mainly of peptidoglycan linked to other hydrophobic compounds, like teichoic acid and proteins, this hydrophobic layer may facilitate the entry of hydrophobic molecules, in turn disrupting the cytoplasmic membrane, thereby enhancing their antibacterial effectiveness (Nazzaro et al., 2013). However, EOs are complex mixtures of compounds and show a great diversity in qualitative and quantitative composition dependent on factors such as the plant organ, age, vegetative state, climate, soil composition, and the extraction method. Each of these constituents' mode of action and the interactions—whether antagonistic, additive, or synergistic—determine how effective EO is against microorganisms. This extreme variability of the EO chemical profile and the ratio of its constituents has frequently resulted in different activities, even within the same contexts. Furthermore, there are several modes of action by which EOs may exert their antimicrobial effects (Figure 6).

Guimaraes et al. (2019) reported that free terpenes, which in general are highly hydrophobic and are typically abundant in EOs, have powerful antibacterial effects against Gram-negative bacteria. The blockage action of EOs during the stages of duplex DNA transcription into mRNA and mRNA translation for protein synthesis, which affects bacterial DNA replication, is an alternative pathway (Xu et al., 2016). Clove (*Syzygium aromaticum*) spice increases external ATP levels and decreases ATPase activity when used in bactericidal quantities (Cui et al., 2018). Some EOs exhibit antibiofilm effects. Biofilms produced by 3 Gram-negative pathogens (*S. enterica, P. aeruginosa*, and *E. coli*) were reduced by 80% when treated with extracts of the Asian medicinal plants *Andrographis paniculata* and *Holarrhena antidysenterica* at 50 g/mL. This reduction was attributed to bacterial cell membrane damage caused by cinnamaldehyde in these EOs (Masak et al., 2014; Tanwar et al., 2016). In a recent study, carvacrol (**CAR**) and thymol (**THY**) were encapsulated in monolayer (**ML**) and layer-by-layer nanocapsules as part of a novel antibiofilm technique. The researchers discovered that following a 30-min exposure to ML-CAR and ML-THY, *L. innocua* biofilms were reduced by 2.2 log and 2.8 log on the SS surface, respectively (Yammine et al., 2023). In a different study, strategies for controlling *S. aureus* biofilms on polystyrene surfaces based on single and combined applications of plant EOs were assessed. *S. aureus* was particularly sensitive to *Lippia sidoides* oil, but concentrations greater than 2.75% (v/v) were necessary for the complete elimination of 24-h-old biofilm. Furthermore, binary combinations of thyme (*Thymus vulgaris*), *Pimenta pseudochariophyllus*, and *Lip. sidoides* allowed for a considerable reduction of biofilm cells by 4 log CFU/cm^2^ (Vázquez-Sánchez et al., 2018). It thus follows from the above discussion that EO could be used to control biofilm formation in the meat industry and provide a solution for the impending antimicrobial challenge.

Consumer acceptance of sensory changes is usually influenced by their culinary and personal experiences, as food preferences are formed during childhood and vary from person to person. Appearance provides the customers' first impression of the food. In the context of meat, the loss of redness caused by the formation of metmyoglobin negatively affects the appearance of meat. When EOs are directly included as preservatives in meat and meat products, the sensory (physical and chemical) properties are altered due to the lack of inertness of EOs, such as odor and taste (Ruiz-Hernandez et al., 2021). The interactions of EO components with the meat matrix are reportedly the greatest obstacles to EO usage (Hyldgaard et al., 2012). Both the extrinsic properties of the meat matrix in terms of microbial density, microorganism type, atmospheric gaseous composition, and temperature, as well as the intrinsic properties of the meat matrix in terms of fat, protein, water activity, salt concentration, and pH can potentially affect the functionality of EO (Hyldgaard et al., 2012; Prakash et al., 2018). Maintaining the overall efficacy of EOs and their active components to maximize product quality can affect the sensory properties of meat and meat products (Šojić et al., 2019; Pateiro et al., 2021). Thus, one of the main challenges facing the use of EOs as natural preservatives is thought to be the advancement of contemporary technologies for integrating EO into complex food systems in a way that minimizes sensory disruptions and improves meat quality.

### Bacteriophages

**Figure 7:**
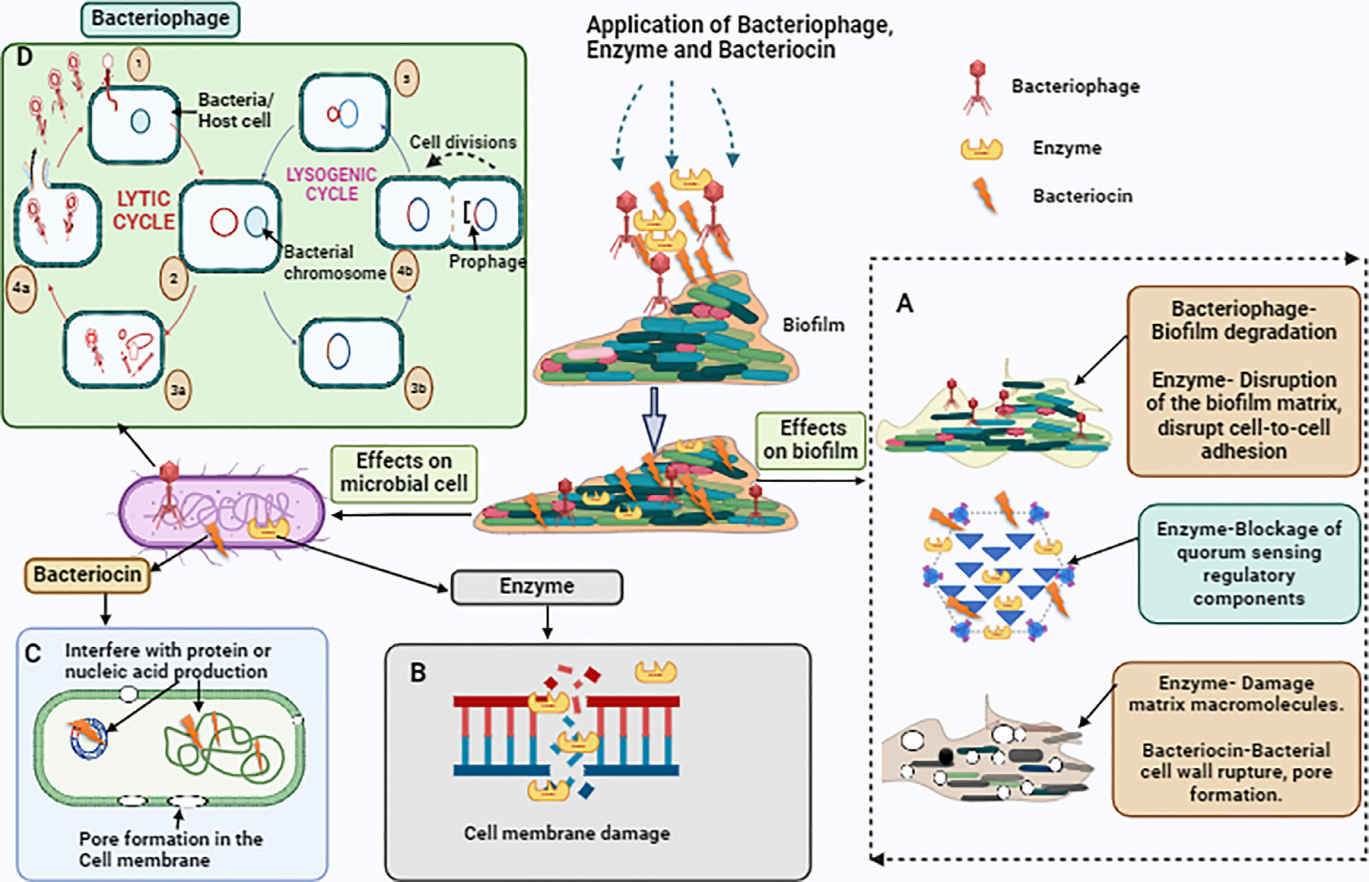
Hypothetical image of biofilm inhibition by bacteriophages, enzymes, and bacteriocins. (A) Disruption of the biofilm matrix, blockage of QS, and pore formation by bacteriophages, enzymes, and bacteriocins. (B) Cell membrane damage by enzymes. (C) Interference with protein or nucleic acid production and pore formation in the cell membrane by bacteriocins. (D) Effects of bacteriophages on host cell/bacteria. (1) Phage attached to host cell. (2) Phage DNA circularizes and enters lytic or lysogenic cycles. (3a) Virions with new phage DNA and proteins. (3b) Phage DNA combines within the bacterial chromosome. (4a) Cell lysis releasing virions. (4b) Lysogenic bacterium reproduces.

Bacteriophages (phages) are common viruses that attack and kill specific bacterial cells while remaining nontoxic to nonspecific bacteria, people, animals, and the environment (Endersen and Coffey, 2020). This specificity results from the selective interaction between the viral receptor and ligand on the bacterial cell surface. Dowah and Clokie (2018) reported that phages can use certain proteins, polysaccharides, lipopolysaccharides, and carbohydrate moieties, along with surface proteins, pili, and flagella, to penetrate bacteria. Phages can follow 2 distinct pathways after infection of bacteria: the lytic cycle or the lysogenic cycle (Figure 7). A phage that follows a lytic cycle is termed virulent (Abedon et al., 2017). In the lytic pathway, the phage's genetic material is duplicated using the bacterium's molecular machinery once the phage has reached the appropriate host. Novel virions are combined and released into the environment immediately after the entrance of the phage. This series of molecular events gradually promote bacterial cell destruction (Abdelsattar et al., 2021). Temperate phages favor the lysogenic cycle. As part of the lysogenic cycle, the virus's nucleic acid fuses with the bacterial genome and multiplies alongside the host as a prophage, thus allowing the phage to return to the lytic cycle in response to unfavorable changes in its surroundings (Sharma et al., 2017).

There is a significant body of work on the application of bacteriophages to control Gram-positive and Gram-negative bacteria in diverse food production settings (O'Sullivan et al., 2019). Several studies have described the effects of bacteriophages against different biofilm-forming bacteria found in meat and on surfaces within meat-processing environments. According to Montso et al. (2021), individual phages can significantly (*P* < 0.05) prevent biofilm formation of *E. coli* on a polystyrene plate surface. When incubated at 25°C for 24 h, the majority of the isolates (80%) did not produce biofilms in the presence of the phages vB_EcoM_10C3, vB_EcoM_10C2, and vB_EcoM_366V; only 20% of the isolates were able to generate weak and moderate biofilms. In another investigation, Shiga toxigenic *E. coli* (**STEC**) O145 cells in biofilms were easily transmitted from the surface of the SS coupon to the beef surface (3.6 log CFU/coupon) even after only 10 s of contact. However, the transfer of biofilm cells from the SS coupons to the beef surface was decreased (*P* < 0.01) by 3.1 log CFU if the STEC O145 cells were grown for 48 h before treatment with phage (2 × 10^10^ PFU/mL at 24°C) (Wang et al., 2020).

A newly discovered *L. monocytogenes* phage named vB-LmoM-SH3-3 has been identified at a food processing facility and demonstrated remarkable reduction biofilm efficacy against *L. monocytogenes* biofilm formed on polystyrene surfaces that come into contact with meat. The researchers observed a reduction of the OD_595_ value from 0.55 to 0.17 at the concentration of 10^6^ PFU/mL phage SH3-3, indicating significant inhibition of biofilm formation after 48 h incubation. Moreover, when the phage concentration was increased to 10^10^ PFU/mL, the OD_595_ value was reduced to 0.07, resulting in complete inhibition of biofilm formation (Zhou et al., 2020).

Figueiredo and Almeida (2017) assessed the single and combined effects of nisin, sodium lactate, and bacteriophage P100 in preventing *L. monocytogenes* growth in slices of ready-to-eat pork ham. After the samples were inoculated with a variety of *L. monocytogenes* strains, their surfaces were treated with the antimicrobials. The *L. monocytogenes* population was reduced to undetectable levels 72 h after contamination by bacteriophage P100.

Exopolysaccharide depolymerase enzymes are found in certain phages, and they facilitate phage invasion and dispersion through the biofilm undergoing treatment (Parasion et al., 2014; Pires et al., 2016). The ability of endolysins and virion-associated peptidoglycan hydrolases to efficiently enter biofilms has led to their evaluation as biofilm-elimination agents (Shen et al., 2013).

Nevertheless, there are some considerations with the use of phages in the food industry. Galie et al. (2018) reported some unfavorable conditions, such as the interactions between phages and conventional sanitizers, as well as unideal temperatures for phages that are frequently used in the food business, which may not be optimal for phages. An additional relevant concern involves the effect of these phages on mixed biofilms formed of various species, which are frequently found on surfaces in the meat industry. Although several phage-based products have been commercialized and approved for use in the United States, there are no regulations or safeguards to date to track bacteriophages and their human intake (Gomez-Gomez et al., 2019). It is widely acknowledged that the emergence of phage-resistant bacteria and a limited range of phage activity could significantly restrict the widespread use of phages (Hyla et al., 2022). Unlike many antibiotics, phages can attack bacteria in biofilms while remaining unaffected by the mechanisms that cause antimicrobial resistance (Pires et al., 2011). Nevertheless, these drawbacks can be overcome by using phage cocktails for various specificities. Resultantly, the lytic spectrum of phage-susceptible bacteria will be expanded such that bacteria will tend to be vulnerable to other phages in the cocktail despite developing resistance toward one of the phages (Ormala and Jalasvuori, 2013; Wang and Zhao, 2022). Furthermore, research should be conducted to formulate strategies for controlling the spread of phage-resistant bacteria.

However, bacteriophage-based techniques for eliminating and destroying biofilms have potential as a safe and environmentally friendly control method. Recent developments in genetic engineering have made it possible to create bacteriophages with desired characteristics, increasing their host range and improving their ability to remove biofilm communities. Based on the above reports, it has been suggested that bacteriophages can be employed to control undesirable biofilms in the meat industry. However, it is essential to contemplate the means of enhancing bacteriophage biosynthesis and implementing it on a commercial scale.

### Enzymes

Enzymes are natural catalysts that can accelerate chemical processes without utilizing anything or being utilized in the process (Chen and Arnold, 2020). Enzymes have been tested for their activity against both single- and multi-species biofilms (Puga et al., 2018). Bacterial cells located within the matrix can be targeted by antibiofilm enzymes, which induce cell lysis (Figure 7). Moreover, EPS is an additional target of enzymes that disrupt cell-to-cell adhesion, separate cells repeatedly, damage matrix macromolecules, and disintegrate the target biofilm (Coughlan et al., 2016). Furthermore, enzymes potentially damage cell membranes, alter intracellular materials, and interfere with QS-mediated communication mechanisms by obstructing the availability of acylated homoserine lactone (**AHL**) (Meireles et al., 2016; Baidamshina et al., 2017; Nahar et al., 2018). Numerous factors affect an enzyme's catalytic activity, including the availability of stimulating or suppressing chemicals, enzyme quantity, media and substrate types, temperature, and pH (Hobbs et al., 2013; Arcus et al., 2016; Arsalan and Younus, 2018). The amylase enzyme of *Bacillus cereus* reduced *S. aureus*-biofilm growth by 81%, 95%, and 100% at concentrations of 40, 45, and 50 µL/mL, respectively (Vaikundamoorthy et al., 2018).

Sanitation practices used in the food industry are frequently insufficient in preventing or controlling the spread of *L. monocytogenes* biofilms. Nguyen and Burrows (2014) observed that the addition of DNase (a DNA-degrading enzyme) during biofilm formation decreased biofilm adhesion to polystyrene by approximately 50% relative to the control. Biofilms that had been developed for 72 h before treatment with DNase were incompletely dispersed after 24 h treatment with DNase (100 g/mL), with approximately 25% of the biofilms remaining undispersed. In contrast, exposure to proteinase K (100 g/mL) completely prevented biofilm development and entirely dispersed the biofilms that had been produced under the same above-mentioned conditions. The combined DNase I and eugenol treatment resulted in a 4.4 log biofilm reduction of *S.* Enteritidis on polyethylene terephthalate (**PET**) and SS surfaces, respectively, within 30 min, which was significantly higher than the individual treatments (*P* < 0.05). Biofilm reductions on PET and smoked duck surfaces were visually confirmed with field emission scanning electron microscopy (Kim et al., 2023). Moreover, a reduction of more than 2 log was achieved on sterilized duck surfaces after combined treatment with 4X minimum inhibitory concentration (**MIC**) of eugenol and DNase 1. The researchers suggested that co-treatment with DNase I and eugenol could be a promising approach to control *S*. Enteritidis biofilms and minimize related health risks from the duck processing plant. In another study, sequential treatment of ficin and PAA led to *S.* Thompson biofilm reductions of 3.2 log, 5.0 log, and 6.5 log CFU/cm^2^ from chicken skin, eggshell, and plastic, respectively (Nahar et al., 2022).

Alginate, a crucial EPS component of the *P. aeruginosa* biofilm, is responsible for surface-attachment stability and capability. Alginate lyase effectively decomposes the alginate in the *P. aeruginosa* biofilm. Li et al. (2019b) created Aly08 chitosan nanoparticles (**AL-LMW-CS-NP**s) by trapping alginate lyase Aly08 on low-molecular-weight chitosan nanoparticles. The resultant immobilization of the low-molecular-weight chitosan nanoparticles enhanced their thermal stability and antibiofilm activity. Moreover, the efficacy of the immobilized AL-LMW-CS-NPs in interrupting the existing mature *P. aeruginosa* biofilm and suppressing biofilm formation exceeded that of free Aly08, which was visualized using a confocal microscope. Furthermore, the sensitivity of *P. aeruginosa* to antibiotics improved substantially owing to biofilm breakdown.

Lysozyme is attractive as a natural preservative in meat and meat products as it shows antibacterial activity against Gram-positive and Gram-negative bacteria (Chung and Hancock, 2000). It is applied in meat products in combination with other antimicrobial agents and hurdles to reduce the concentrations needed to inhibit bacteria. Using a combination of lysozyme (at MIC of 50 mg/L against *Lactobacillus brevis*) and ultra high-pressure homogenization (150-170 MPa) led to a reduction of 6 log in model systems (Tribst et al., 2008). Cannarsi et al. (2008) showed that lysozyme, along with 2% EDTA, could pathogenic microorganisms and extend the shelf life of fresh buffalo meat. In another study, modified lysozyme in heat treated samples has been shown to exhibit higher antibacterial activity in ground pork (Cegielska-Radziejewska and Szablewski, 2013).

As there remain few reports on the antibiofilm activity of enzymes in meat and meat products, more investigations are required to determine the approaches to achieve better application of enzymes in meat products conforming to market trends. The use of several enzymes in the food industry has been authorized to eliminate various microorganisms in food products. Interestingly, the effectiveness of enzymes as antimicrobial agents does not compromise the safety of food products (D'Angelo et al., 2018). Moreover, enzymatic removal of biofilm is a green technique that has no known negative effects on the environment. According to our literature review, the most effective methods for the complete elimination of biofilm have involved by applying various enzymes, either alone or combined with other technologies. Nevertheless, the industrial application of all these enzymatic alternatives is still hampered due to the huge expense of these treatments.

### Bacteriocins

Bacteriocins are antimicrobial proteins produced by Gram-positive and Gram-negative bacteria, particularly lactic acid bacteria (**LAB**), and they are frequently used in food-preservation processes (Camargo et al., 2018). A few bacteriocins interfere with protein or nucleic acid production, whereas others often act by inducing pore formation in the target cell membrane or impeding cell wall formation (Figure 7) (Meade et al., 2020; Negash and Tsehai, 2020; Soltani et al., 2021). The bacteriocins that have been examined extensively for use in meat and meat products include nisin, pediocin, and sakacin (Xu et al., 2022). Studies that have examined the antimicrobial effects of bacteriocins have typically used either pure products, such as nisin- and pediocin-containing products, or partially purified forms, such as the cell-free supernatants of bacteriocin-producing bacteria. Fresh meat is often treated using bacteriocin solutions that have been prepared to achieve a specific antibacterial activity by volume or weight.

Bacteriocins can inhibit the biofilm formation of Gram-negative bacteria. According to Seo and Kang (2020), treatment with bacteriocins purified from *Pediococcus acidilactici* K10 (bacteriocin K10) and HW01 (bacteriocin HW01) significantly reduced the amount of *S*. Typhimurium biofilm on SS surfaces and chicken meat. Bacteriocin K10 treatment decreased *S.* Typhimurium biofilm formation by approximately 2.0 log CFU per SS coupon after 72 h. When applied to chicken meat, bacteriocin K10 (*P* < 0.05) reduced the *S*. Typhimurium biofilm by nearly 50% after 72 h of incubation. Moreover, bacteriocin K10 and HW01 inhibited an existing *S*. Typhimurium biofilm after 72 h of incubation (*P* < 0.05), which indicated that both bacteriocins successfully eradicated the biofilm from chicken meat. In a different study, *L. lactis* UQ2 was observed to reduce > 5 log of *L. monocytogenes* Scott A adhering to SS (García-Almendárez et al., 2008).

The effectiveness of bacteriocins in inhibiting bacterial colonization, particularly those produced by Generally Recognized as Safe (**GRAS**) microorganisms, such as LAB, has been studied thoroughly. These bacteriocins include pediocins, lactocins, and garvicin (Castellano et al., 2017). Their use poses no risk when they interact with animal tissues, and their commercial application should not cause any serious problems (Strempel et al., 2015; Galie et al., 2018). The applications of those bacteriocins that have been approved for commercial use or have been granted GRAS status, research aimed at finding more effective ways to use bacteriocins, particularly as part of hurdle technology, will speed up the industrial-scale uses of bacteriocins for the control of biofilm in the meat industry.

## FUTURE PERSPECTIVES

In meat products, biofilm formation is a major and ongoing concern because it potentially leads to meat spoilage, foodborne illnesses, and financial loss. Owing to its resistance to various inhibitory techniques, biofilm is extremely challenging to eradicate or control. Although various physical, chemical, and biological methods have been used, significant limitations and challenges have persisted, such as functional impairment, an inappropriate interface-region temperature, cost, safety concerns, and legal and regulatory dilemmas. Partial elimination of biofilm from surfaces and food-contact equipment can be accomplished using the methods documented in this paper, including physical, chemical, and biological exposure. Future studies that focus on developing effective control techniques in real-world applications while minimizing the drawbacks and economic impediments associated with existing methods are warranted.

## CONCLUSION

A recent development in the field of food safety is the elimination of foodborne pathogens and biofilm formation during the processing of meat and meat products. Bacterial biofilms associated with meat and meat products have been addressed. However, significant challenges related to preventing biofilm formation in the meat industry remain. The majority of the existing sanitization techniques use chemical disinfectants. However, these sanitizers cannot eliminate mature biofilms from food-contact surfaces and have limited effects on biofilms, even after prolonged exposure times. Therefore, in addition to safe and effective sanitizing treatments, practical and affordable strategies are required to control, eliminate, and prevent biofilm formation in the meat industry. In this study, we extensively reviewed advanced information and cutting-edge technologies currently utilized for microbial reduction and biofilm elimination in meat and meat products. In terms of chemical implementation, microbial loads on meats were significantly reduced by using EOs. Bacteriophage cocktails proved more efficient at reducing bacterial biofilms than individual bacteriophages. Ozone gas and US are potentially useful as additional cleansing measures; notwithstanding, investigations on their use have not demonstrated their ability to suppress biofilm effectively. Moreover, biofilm could be effectively destroyed by bacteriocins and CP. Therefore, we recommend that, along with the existing cleansing and antibiotic treatment practices, biological measures, such as a combination of EO and phage, be used extensively in industrial applications to reduce biofilm formation.

## DISCLOSURES

The authors declare no conflicts of Interest.

## Appendix Supplementary materials

Image, application 1
